# Differential Expression of Arabinogalactan in Response to Inclination in Stem of *Pinus radiata* Seedlings

**DOI:** 10.3390/plants11091190

**Published:** 2022-04-28

**Authors:** Tamara Méndez, Yazmina Stappung, María A. Moya-León, Raúl Herrera

**Affiliations:** Instituto de Ciencias Biológicas, Universidad de Talca, Av. Lircay s/n, Talca 3465548, Chile; tamendez@utalca.cl (T.M.); ystappung@utalca.cl (Y.S.); alemoya@utalca.cl (M.A.M.-L.)

**Keywords:** AGPs, fasciclin-like, compression wood, *Pinus radiata*

## Abstract

Arabinogalactan proteins (AGPs) are members of a family of proteins that play important roles in cell wall dynamics. AGPs from inclined pines were determined using JIM7, LM2, and LM6 antibodies, showing a higher concentration in one side of the stem. The accumulation of AGPs in xylem and cell wall tissues is enhanced in response to loss of tree stem verticality. The differential gene expression of AGPs indicates that these proteins could be involved in the early response to inclination and also trigger signals such as lignin accumulation, as well as thicken cell wall and lamella media to restore stem vertical growth. A subfamily member of AGPs, which is Fasciclin-like has been described in angiosperm species as inducing tension wood and in some gymnosperms. A search for gene sequences of this subfamily was performed on an RNA-seq library, where 12 sequences were identified containing one or two fasciclin I domains (FAS), named PrFLA1 to PrFLA12. Four of these sequences were phylogenetically classified in group A, where PrFLA1 and PrFLA4 are differentially expressed in tilted pine trees.

## 1. Introduction

Arabinogalactan proteins (AGPs) are a complex and highly glycosylated superfamily present in cell walls, the surface of plasma membranes and extracellular secretions [1]. These proteins are composed of a protein nucleus linked to one or more O-glycosylated amino acids by β→ 1–3 or β→ 1–6-galactan chains to other sugars, mostly arabinose, including glucuronic acid [2,3]. The AGPs linked to the glycosylphosphatidylinositol (GPI) anchor signal in the C-terminal and have also been found on the plasma membrane [4]. Classical AGPs usually have N-terminal signal peptide, are rich in hydroxyproline, and possess a central domain called PAST-rich, which is rich in Pro, Ala, Ser, and Thr. The PAST-rich domain is usually separated if more than one Lys-rich region is present. Six different proteins can be recognized based in the protein structure, which include classical type, rich in lysine, AG peptides, fasciclin-like (FLAs), non-classical and chimeras [5].

Classical AGPs present domains rich in hydroxyproline, alanine, serine, threonine and glycine, while non-classical domains have hydroxyproline-poor domains rich in asparagine or cysteine. A signal for the link to a GPI at the C-terminal domain is present in the classical AGPs and absent in the non-classical AGPs. From a structural point of view, the function of the GPI allows binding of the AGPs to the plasma membrane [6]. However, the AG peptides contain short backbones [7]. Non-classical AGPs have hydroxyproline-poor domains and are rich in cysteine or asparagine and do not have a C-terminal GPI domain, so from a structural point of view, since they lack GPI, they are found as soluble molecules on the surface of the cell wall [8]. The chimeric type AGPs contain long polysaccharides arabinogalactans of type II AGPs and short hydroxyproline-oligoarabinosides of extensins [9].

Fasciclin proteins have one or two fasciclin I domains (FAS1), which play an important role in cellular adhesion [7,10]. The two regions are highly conserved and have nine amino acids each. The H1 region is composed of Leu-Thr-Ile/Leu-Phe-Ala/Val and Pro. The H2 region is composed of Val/Leu-Ile/Val-Ile-Tyr-Gln/Glu-Val-Asp/Asn-Lys. A less conserved but central YH motif has also been found, which includes the amino acids Leu-Leu/Ile/Cys/Val-Leu/Cys-Phe-Tyr-His-Ala/Ile-Leu and Pro [11,12]. Moreover, these proteins can be subclassified into four different groups (A, B, C and D), depending in the number of FAS domains present in the protein. In addition, an anchoring site may or may not have a GPI section in the C-terminal sector [13]. It has been suggested that this subclass of AGP is implicated in plant growth, development and response to abiotic stress [5].

AGPs have been attributed to have roles in plant growth and development, both structurally and regulatory, from root elongation, somatic embryogenesis, response to hormones, xylem differentiation [14,15,16], growth and direction of the pollen tube [17], programmed cell death, cell expansion and tolerance to saline stress, among others, as we;; as host-pathogen interaction (response to abiotic stress) and cellular signaling [6]. These proteins are present from plasma membrane to the extracellular matrix of plants [18] and are integral to many adhesion-based mechanisms [19].

The characteristic structure of AGPs has been described in conifers [20], and their functional role was reported from *Pinus taeda* [21]. Two AGPs (PtX3H6 and PtX14A9) were differentially expressed in xylem. The PtX14A9 gene is expressed mostly in radial expansion of seedlings hypocotyl, suggesting a role during seedling development. The differences in expression of both genes are due to hormonal signals. PtX14A9 is a probable ortholog of FLA11. Additionally, PtaAGP3 and PtaAGP6 were found to be differentially expressed in xylem tissue, associated with secondary cell wall formation, xylem differentiation, and wood formation [21]. At the same time, PtaAGP6 is highly expressed in immature xylem in vertical or normal wood, as well as in compression wood [21]. Furthermore, PtaAGP4 is expressed mostly in compression wood xylem at the lower stem side [22]. Additionally, Ptx3H6 and Ptx14A9 are expressed preferentially in xylem, in comparison to other tissues.

Compression wood (CW) is formed in gymnosperm in response to trunk vertical loss, inducing eccentric stem growth with a greater proportion of lignin, rounded tracheids, absence of wall S3 and greater fibrillar angles in wall S2, the presence of intercellular spaces, and a reduction in the proportion of lignin in the middle lamina [23,24]. Trunk deformation decreases wood quality and affects the production of pulp and paper [25]. This type of wood is formed in conifers in the lower side of tree trunk and in branches in response to non-vertical orientation associated with initial gravitropic stress [26].

Angiosperm species react to loss of trunk verticality by inducing tension wood (TW). A double mutant for *FLA* in *Arabidopsis thaliana* (atfla11/atfla12) showed altered mechanical properties of its secondary cell wall-rich stems, and the chemical composition and cell wall structure showed a reduction in galactose, arabinose and cellulose, and a concomitant increase in lignin content [13]. The authors speculate that FLA proteins, through their FAS1 domains, might form a heteromeric higher-order network, strengthening the interaction between cellulose microfibrils. In *Populus tormentosa, PtFLA6* was abundantly expressed in TW and localized in differentiating G-fibers. When this gene was silenced with antisense RNA, a reduction in *PtFLA6*, as well as in other *FLA* genes, was observed [27,28]. The stem biomechanics was altered in a transgenic tree due to a reduction in the composition of lignin and cellulose. The role for these FLA proteins in the recovery of trunk verticality can therefore be assumed.

In radiata pine, some FLA nucleotides sequences were described, mainly in samples from xylem in compression wood. However, when all the ESTs reported from Li [29,30] were checked, mostly incomplete or misidentified proteins were found. In this work, a comparison between *FLA* genes within a RNA-seq data prepared from inclined radiata pine seedlings is reported, in addition to phylogenetic analysis. Stem histological preparations were prepared to perform phenotypic characterization using monoclonal antibodies to identify AGP and the qRT-PCR technique was used to validate the differential expression for different genes found in pine trees.

## 2. Results

### 2.1. Molecular and Biochemical Description of Pine AGP

A list of 102 ESTs related to AGPs was found from *Pinus radiata* D. Don cDNA xylem libraries, which included the data from the cell wall protein categorization of the Maize wall data base. Those EST were distributed as 73 ESTs, matched to AGP, 2 to extensions, 11 to HGRP and 16 to PRP. The radiata pine RNA-seq library from the bioproject carried out by Li et al. was examined to obtain full sequences and the FAS domain within those genes [29,30]. When the RNAseq library prepared in our laboratory was examined, a list of 12 sequences were obtained, all of which presented the conserved domains. Even though a larger number of sequences was found and reported within the fasciclin domain, only those within the FAS domain and/or PAST-rich domain were considered, resulting in 12 sequences, which were further analyzed.

**Figure 1:**
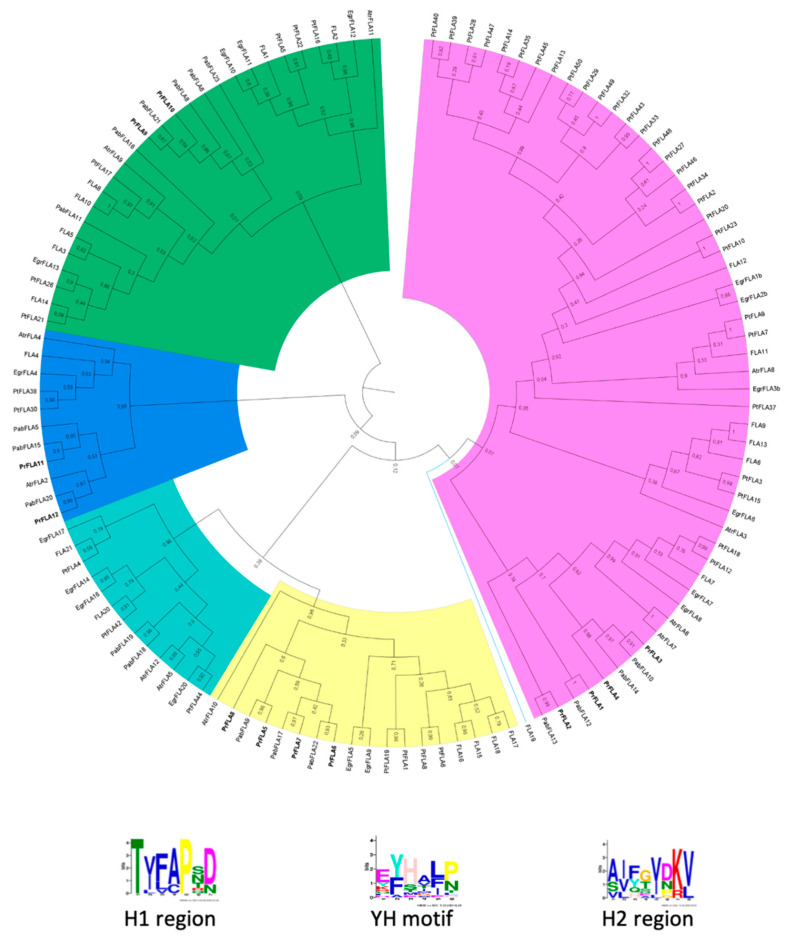
Phylogenetic comparison of pine protein sequences with Arabidopsis, Amborella, Eucalyptus, Populus and Picea. Amino acid sequences of fasciclin were aligned by ClustalX, and the phylogenetic tree was built by MEGA X using Maximum Likelihood (ML) and JTT matrix-based model with 5000 N° of Bootstrap replications. The tree was divided into five major clades: Group A (pink), Group B (yellow), Group C (green), Group D1 and Group D2. The conserved motifs (H1, H2, and YH motifs) in pine’s sequences shown below the tree were found using the MEME web server.

A phylogenetic tree was constructed using these twelve sequences from radiata pine in addition to the other 126 AGP sequences (Supplementary Table S1) from *Amborella trichopoda* (12), *Eucaliptus grandis* (19)*, Populus trichocarpa* (49)*, Arabidopsis thaliana* (22)*,* and *Picea abies* (24). Five cluster groups were generated in the phylogenetic analysis (Figure 1). Group A is the larger cluster group with 53 genes and includes *FLA6*, *FLA7*, *FLA9*, *FLA11*, *FLA12* and *FLA13* from *A. thaliana*. Four radiata pine genes classified in group A: *PrPLA1*, *PrPLA2*, *PrPLA3* and *PrPLA4*. *PrFLA1* were clustered next to *PabFLA12*, *PrFLA2* next to *PabFLA13*, at the same time *PrFLA3* grouped next to *PabFLA10*, and *PrFLA4,* in the same clade next to *PabFLA14* and *PabFLA10*.

In the case of group B, all sequences were similar to AtFLA17 40 with a minimum of 60% identity. Group B clustered 18 sequences and includes four radiata pine genes: *PrPLA5* to *PrPLA8*. *PrFLA5* grouped together with *PabFLA9*, *PrFLA6* with *PabFLA22*, *PrFLA7* with *PabFLA17*, and *PrFLA8* shares the same root with the *PrFLA5–7*.

Group C contains 27 sequences divided in two large groups, where *PrFLA9* and *PrFLA10* were grouped together, as well as *PabFLA21*, *PabFLA8*, *PabFLA6*, and *PabFLA23*. The sequences showed similarity with *AtFLA8* (Table 1).

Finally, two D groups, named D1–D2, were identified (Figure 1). D1 (light blue) was not included in any PrFLA sequence, but in the D2 group (blue), two sequences were clustered: *PrFLA11* and *12*. *PrFLA11* next to *PabFLA15*, and *PrFLA12* next to *PabFLA20*, in a group of eleven sequences.

All twelve *PrFLA* sequences were examined for consensus conserved regions H1, YH, and H2 using MEME.

**Figure 2:**
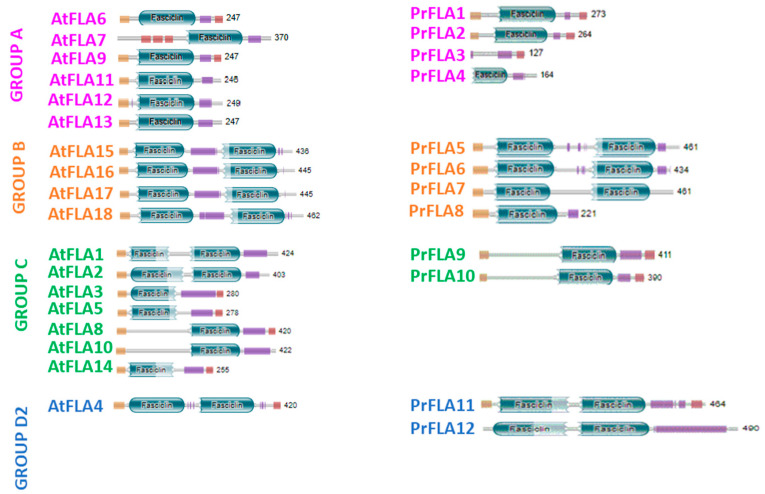
Analysis of domains and conserved motifs within FAS proteins identified in radiata pine in comparison with *A. thaliana* sequences. The search for domains and conserved motifs was performed with Pfam. The signal peptide is indicated in yellow, green capsules correspond to Fasciclin domains, binding to transmembrane (TM and signal peptide) is indicated in red, could be GPI anchor, and Disorder or PA motifs of AGP are indicated in lilac.

The four protein sequences from radiata pine (*PrFLA1* to *PrFLA4*) which were classified in group A shared an identity between 52 and 46% with *AtFLA6* (Table 1) and displayed one fasciclin domain (Figure 2). The H2 motif was more conserved within the FAS domain when the pine sequences were compared against Arabidopsis (Supplementary Figure S1). When the four sequences were analyzed using HmmScan, two protein sequences (*PrFLA1* and *PrFLA2*) included a signal peptide, but only *PrFLA2* displayed this sequence when SignalP was employed. Furthermore, when big-PI Plant Predictor [31] was used, three of the predicted *PrFLAs* from group A showed the GPI anchor (Figure 2).

Fasciclin-like proteins from group B were mainly defined according to the Arabidopsis nomenclature for the presence of two FAS domains separated by a PAST motif, but they do not have a GPI anchor. The four pine sequences belonging to this group (*PrFLA5* to *PrFLA8*) showed high sequence similarity with *AtFLA17* (Table 1). Three out of four pine sequences present all relevant domains (regions H1, H2 and motif YH; Supplementary Figure S2), but *PrFLA8* contains only one FAS domain (Figure 2).

*PrFLA9* and *PrFLA10* were classified in group C. Both sequences showed similarity with *AtFLA8* (Table 1). In this case, these proteins can have 1 or 2 FAS domains, in some cases if FLA protein has 1 FAS domain, it could be at the amino or carboxi end (Figure 2). When *PrFLA* 9/10 to *AtFLA*1/2/3/5/8/10/14 were compared, all proteins had the FAS domain at the carboxi end (Supplementary Figure S3).

Finally, *PrFLA11* and *PrFLA12* were included in group D2, and shared high similarity with the AtFLA4 sequence (Figure 2). Both radiata pine sequences showed two FAS domains, and the second FAS domain was more conserved than the first one (Supplementary Figure S4).

### 2.2. Determination of Total AGP and Identification of Differential Epitopes

The presence of the AGP domain was evaluated using two strategies. The first based was based on the reaction between AGP and Yariv reagent, and the second used the antibodies JIM7, LM2 and LM6. The antibody JIM7 binds homogalacturonan and recognizes specific methyl esterification patterns [32]. LM2 and LM6 bind different AGP-antibodies, LM2 recognizes (1→6)-β-D-Galp units with terminal ß-D-GlcAp in AGP. LM6 recognizes (1→5)-α-L-Araf oligomers in arabinan or AGP [32,33].

**Figure 3:**
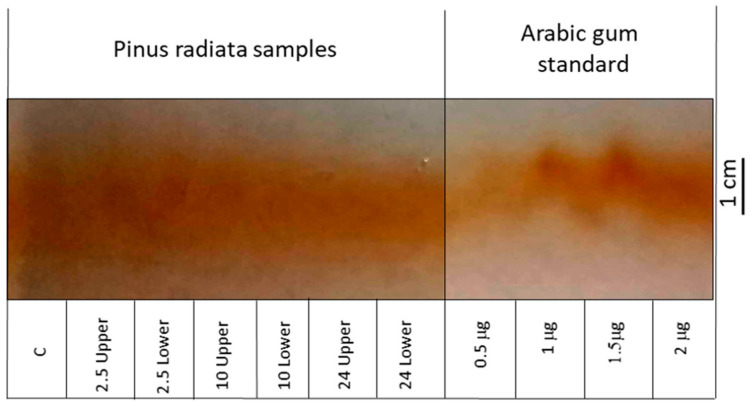
Rocket electrophoresis of pine stem seedlings samples obtained after different inclination times (2.5 h, 10 h, 24 h) from the lower side or upper side of bent stems. C, control sample (not inclined, 0 h). Arabic gum was employed as a standard at concentrations of 0.5 μg, 1 μg, 1.5 μg and 2 μg.

The AGP relative abundance was determined using electrophoresis rockets (Figure 3). Stem samples of one year old radiata pine taken from the lower stem side after 10 h of bending and after 2.5 h of bending (lower and upper sides) showed the higher intensity of AGPs in rocket electrophoresis. Arabic gum was used as a standard and the more intense samples showed a concentration of 1 μg.

**Figure 4:**
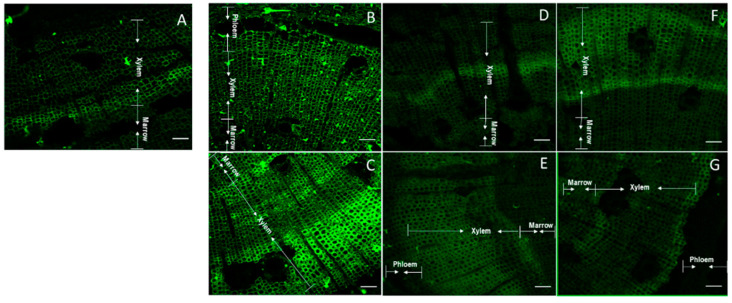
Confocal microscopy of thin sections of one year old radiata pine seedlings incubated with JIM7 antibody and Alexa Fluor. Control stem (non-inclined) (**A**). Upper side of inclined stems after 2.5 h (**B**), 10 h (**D**) and 24 h (**F**). Lower side of inclined stems after 2.5 h (**C**), 10 h (**E**) and 24 h (**G**). Arrows indicate the section within the cuts corresponding to marrow, xylem and phloem. Scale of 100 μm.

A particular stain pattern was observed when JIM7 was used (Figure 4). A clear separation phase was observed in the xylem of control stems (non-tilted) (Figure 4A). A more homogenous luminescence was observed in the upper stem side after 2.5 h of bending (Figure 4B). Nevertheless, the major intensity of the new emerging xylem cells was observed in the inferior stem side after 2.5 h of inclination (Figure 4C). The signal intensity grew covering the new xylem and floem cells after 10 h inclination (Figure 4D,E). However, after 10 h of inclination, the fluorescence of homogalacturonans detected on xylem cells at the upper stem side was less intense (Figure 4D). On the contrary, after 24 h of inclination, the fluorescence of JIM7 was more intense in xylem cells at the upper stem side than in lower stem side (Figure 4F,G). When the staining area was compared with Imagej, the samples from the upper stem sides after 2.5 h of inclination showed a greater staining area compared to inferior stem sides (Supplementary Figure S5A).

**Figure 5:**
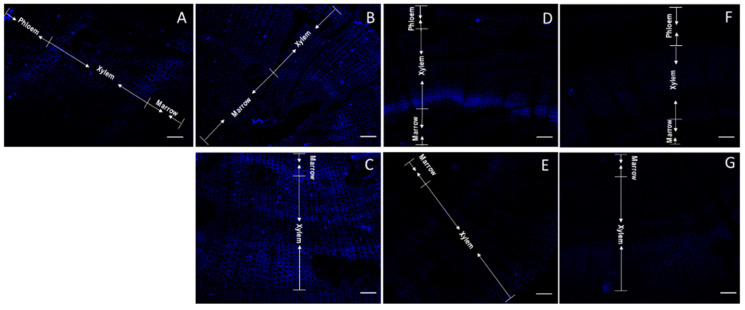
Confocal microscopy of thin sections of one year old radiata pine seedlings incubated with LM2 antibody and Alexa Fluor. Control stem (non-inclined) (**A**). Upper side of inclined stems after 2.5 h (**B**), 10 h (**D**) and 24 h (**F**). Lower side of inclined stems after 2.5 h (**C**), 10 h (**E**) and 24 h (**G**). Arrows indicate the section within the cuts corresponding to marrow, xylem and phloem. Scale of 100 μm.

Arabinogalactan proteins were labeled with LM2 antibody. A slight separation between both marrow and xylem and xylem and floem was observed in non-inclined stems (Figure 5A). A slight major luminescence of arabinogalactan was observed in the lower stem side after 2.5 h of inclination (Figure 5C), contrasting with no changes at 2.5 h in the upper stem side (Figure 5B). On contrary, major arabinogalactans were observed in the upper stem side at 10 and 24 h of inclination (Figure 5D,F). The stem samples taken after 2.5 up and down inclination showed a major stained area but without statistical significance, while the other samples showed a smaller staining area than the control (Supplementary Figure S5B).

**Figure 6:**
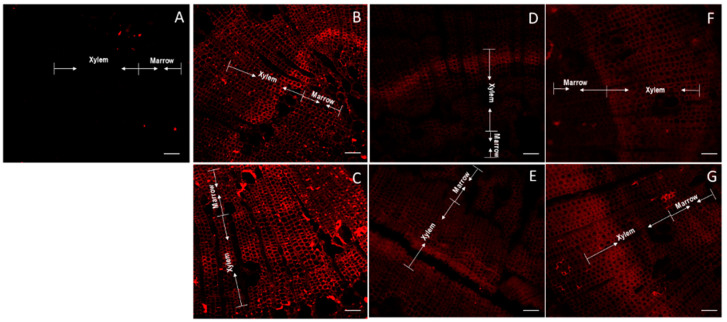
Confocal microscopy of thin sections of one year old radiata pine seedlings incubated with LM6 antibody and Alexa Fluor. Control stem (non-inclined) (**A**). Upper side of inclined stems after 2.5 h (**B**), 10 h (**D**) and 24 h (**F**). Lower side of inclined stems after 2.5 h (**C**), 10 h (**E**) and 24 h (**G**). Arrows indicate the section within the cuts corresponding to marrow, xylem and phloem. Scale of 100 μm.

Then, the third arabino residue ((1→5)-α-L-arabinosilo) was observed using LM6 antibody. A clear separation of marrow and xylem was observed after 2.5 h of inclination (Figure 6B,C), contrasting with the control (Figure 6A). The intense and homogenic adhesion observed at 2.5 h of inclination in the lower stem side slowed down at 10 h (Figure 6D,E). The accumulation of arabino residues in the xylem cells in the upper and lower stem cut, decreases the intensity after 24 h of inclination (Figure 6F,G), even so, all the samples showed a larger staining area than the control, especially at 2.5 h (Supplementary Figure S5C).

Total amounts of proteins were determined by Elisa (data not shown). No significant differences were observed when JIM7 antibody was used (Supplementary Figure S5A). Differences was found when LM2 antibody was used with the control and samples from lower stem side showing a similar value (Supplementary Figure S5B). A peak of absorbance was determined in samples collected from the lower stem side at 2.5 and 10 h and a further decrease at 24 h was observed when LM6 was used (Supplementary Figure S5C).

Finally, samples from the basal and medium cut stem were taken and the relative expression analyzed for four genes associated with group A were compared (Table 2).

**Figure 7:**
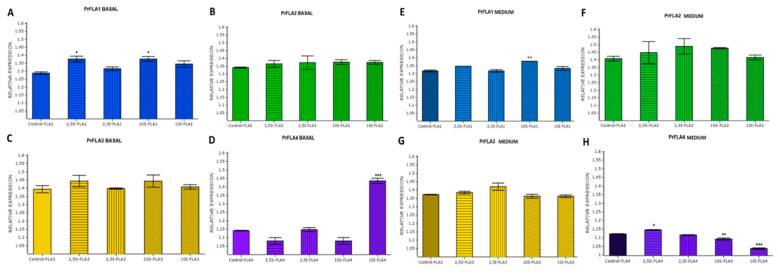
Relative expression of gene from group A in phylogenic. Comparison of basal and medium cut of stem on radiata pine seedlings, at different time and stem side (Control or Time 0, 2.5 h S (upper stem side), 2.5 h I (lower stem side), 10 h S (upper stem side), 10 h I (lower stem side)). (**A**) PrFLA1 relative expression in basal cut samples. (**B**) PrFLA2 relative expression in basal cut samples. (**C**) PrFLA3 relative expression in basal cut samples. (**D**) PrFLA4 relative expression in basal cut samples. (**E**) PrFLA1 relative expression in middle cut samples. (**F**) PrFLA2 relative expression in middle cut samples. (**G**) PrFLA3 relative expression in middle cut samples. (**H**) PrFLA4 relative expression in middle cut samples. Asterisks indicate statistical significance between control and sample at each sampling time (* *p* < 0.05, ** *p* < 0.01, *** *p* < 0.001, Student´s *t*-test).

Relative expression value for PrFLA1 at the base of the stem was significantly high at the upper stem side for 2.5 and 10 h of inclination (Figure 7A). Both genes PrFLA2 (Figure 7B) and PrFLA3 (Figure 7C) did not show statistical differences. Nevertheless, in the case of PrFLA3, the same tendency as PrFLA1 was observed, which means high accumulation of transcripts at 2.5 and 10 h in the upper stem side. On the contrary, a reduction in the accumulation of transcript for PrFLA4 was observed at the upper stem side at 10 h, but an increase in the accumulation of transcript at the lower stem side (Figure 7D).

In the second third of the stem PrFLA1, a slight significantly higher accumulation of transcript was observed at 10 h upper stem side compared to the other time of inclination (Figure 7E). In the case of the genes PrFLA2 (Figure 7F) and PrFLA3 (Figure 7G), no significant differences were observed. On the contrary, a different expression pattern was observed for PrFLA4 (Figure 7H). In this case, an initial higher expression was observed at 2.5 h on the upper stem side, which was reduced with time in both stem sides.

## 3. Discussion

Each of the twelve radiata pine sequences selected in this study were grouped into one of the four main groups described for the fasciclin protein family (groups A-B-C-D) in Figure 2. These groups were defined for the presence of FLA domains and GPI anchor, and showed for proteins in Arabidopsis [7], eucalyptus [13,34], and *Oryza sativa* [35].

Two large clades were observed in group A (Figure 1), in which four sequences of radiata pine were included (PrFLA1, PrFLA2, PrFLA3, PrFLA4), with one FAS domain. These sequences were in the same clade as the gymnosperm *Picea abies* [11], and two *Pinus taeda* sequences (PtFLA18 and PtFLA12), which present 40% PAST motif and a differential expression in xylem [36]. The four pine sequences from this group shared the same length for PAST domain and a similarity between 38% and 48% (Table 1). Even if these pine sequences were more similar to AtFLA7/6, a major resemblance is found with AtFLA11 and AtFLA12 sequences, because these two Arabidopsis sequences were expressed in stems and specifically in sclerenchyma cells (xylem) [13,37]. The sequences belonging to this group are associated with the regulation of the cell wall [13]. In this sense, EgrFLA is involved in the mechanical modification of stems, particularly in microfiber angle for the transition of cellulose enlargement and thickening of the plant cell wall [38].

Group A includes genes such as AtFLA6, AtFLA9 and AtFLA13. It has been reported that AtFLA9 interacts physically with receptor-like kinase (RLK) and both act on cellulose synthase, which suggest that FLA proteins from this group can modulate cellulose biosynthesis and primary cell wall biosynthesis [12]. It has been shown that AtFLA11/12 and PtFLA10 were highly expressed in stems and differentiated xylem [5]. This evidence suggests that these proteins are functionally specific in the formation of woody structures, due to the fact that they were mostly expressed in xylem [12,13,34,36,39]. On the other hand, PtFLA26 (43% PAST) and PtFLA21 (38% PAST) showed higher expression in spring than in winter, and in male catkin tissue [5,36]. It is noticeable that PtrFLA26 is a different gene but used with the same nomenclature in the work of these two authors. The sequences reported by Showalter et al. was used in this work [5,36].

The sequences classified in group A contain one FAS domain, however the outputs of HMM and Pfam databases were contradictory, and, according to the HMM PrFLA3 protein, does not present the FAS domain (Figure 2, Table 1). The analysis of PrFLA3 using ScanProsite showed a zone rich in serine and proline, which continues to be classified as AGP, without a probable AGP motif (Table 1). The H1/YH/H2 regions were incomplete and, possibly, a non-functional protein or without cell adhesion characteristics could be incorporated in one of the databases.

The FLA proteins from group B were more homogeneous, with few members and characterized as having at least two FAS domains and the absence of a GPI anchor. However, PrFLA8 presents only one FAS domain, and the remaining sequences contain the two FAS domains (PrFLA5/6/7). The genes from Arabidopsis were expressed in seeds, embryo tissue, and seed coat [40].

Two sub-clades can be observed in group C, with one or two FAS domains, including EgrFLA10/11/12, and PtFLA22 [34,36]. The gene from *Pinus taeda* is expressed in female inflorescence and presented 33% PAST. Interestingly, PrFLA9 showed similar percentage of PAST sequences, as well as PrFLA10 with 36% PAST, even though these two sequences were clustered on the second clade with *P. abies* PabFLA 6/8/21. From the sequences characterized from this group, AtFLA3 seems to be essential for microspore formation [41,42], it is expressed in pollen grains and tube, possibly by participating in cellulose deposition. Unfortunately, we could not see what happened in flower organs or with the flowering phenomenon, as work had been performed in pine seedlings of one year old without flowers. PabFLA23 sequence was classified in group D1, probably due to the low percentage of PAST sequences [11]. EgrFLA13 has also been reported but no differential expression was observed in the leaves of either stems [34]. Other genes such as AtFLA3/5/8/10 were associated with pollen development and maturation [39,42]. No further functional evidence has been reported for PAST sequences in plants.

Group D is the most diverse clade, which is divided into D1 and D2; however, only two pine sequences were classified in D2 (PrFLA11 and PrFLA12). FLA4 from Arabidopsis is found in the same clade (Figure 1), as it shows characteristics such as tolerance to salt stress and root development [39,41,42], as well as, cell expansion and stress signaling [34].

The Yariv reagent binds specifically to arabinogalactan proteins, and mainly to those proteins located in the cell surface, which has been observed in different plant tissues [15,43]. AGPs isolated from embryonic cells of *Pinus caribaea* showed a high rotation of proteoglycans, allowing cells to react to the environment [44]. In this sense, it is possible that the expression or overexpression of AGP proteins can reorient stem cells to vertical growth. In poplar, a greater amount of AGPs was observed in tension wood compared to opposite wood, during an experiment where poplar trees were inclined with an angle of 45° for two months [45]. In young radiata pine, a high abundance of AGP was found at the early stage of response to inclination.

Total AGPs were determined by a cross electrophoresis, which requires the Yariv reagent [45,46]. A differential accumulation of AGPs in the lower stem side was within the frame of the time course, but the results were not conclusive. In gymnosperm, another method was used based on nitrogen content determination for protein and hydroxyproline, which could be the difference found in our strategy [47]. Genes involved in cell wall remodeling have been reported as differentially expressed when radiata pine seedlings were inclined. The differential expression for *XTH* [48], *Expansins* [49] and genes from the secondary metabolism [50] takes place over time on either stem sides (upper or lower side), in response to stem bending.

AGPs in woody tissues were detected in *Pinus taeda* trees of 11 and 15 years old using JIM13 antibody, showing that AGPs were abundant and specific to differentiated xylem cells and linked to a secondary wall thickening [51]. A demarcated strip corresponding to cells of differentiated xylem was observed because JIM antibodies recognize carbohydrates from AGPs epitopes. Differentiated xylem cells were clearly observed with JIM7, especially in the lower stem side after 2.5 h of bending. JIM7 has been used to identify AGPs in arabidopsis seed mucilage together with LM6 that identifies (1→5)-α-L-arabinans [32]. Most reports for AGPs in plants have been performed in root samples such as barley [52], but few have been used in woody plants cuts, as in the present report.

The analysis of relative expression suggests that genes from group A show a differentially higher accumulation of transcripts in the upper stem side. The gene PrFLA1 shows a greater accumulation of transcript at the base and the middle half of the stem for both times 2.5 y 10 h. Interestingly, the higher concentrations of AGPs using immunolocalization was observed at the lower stem side. Not all members of the gene family were analyzed by qRT-PCR, which may be the reason for the difference observed. However, the AGPs at the lower stem side could likely play a role as a signal transductor [47]. Furthermore, it is important to consider that there is a major accumulation of lignin and an altered amount of microfibrils when AtFLA11/12 is overexpressed in inclined pine seedlings [53].

## 4. Materials and Methods

### 4.1. Sequence Analysis

Putative FLA sequences from radiata pine were identified using “Fasciclin-like” sequences from coniferous and other plant species available from NCBI as a reference for the alignment into transcriptomic data, and the “Finding-AGP” search algorithm [54]. Additionally, RNAseq libraries were built from inclined radiata pine and sequences loaded in GenBank with the name of Bioproject ID PRJNA822053.

An ORF prediction was performed by AUGUSTUS [55], using only full CDS. A comparison was made using the Pfam [56] and PROSITE [57] database to identify previously described critical domains and motifs, respectively. Signal peptides prediction was made using the SignalP 5.0 server [58,59] and transmembrane helices prediction using TMHMM 2.0 server [60]. To identify the GPI-anchored signal, the GPI modification site prediction server BIG-PI was used [31]. Finally, alignment visualization was performed with ESPrit 3.0 [61].

### 4.2. Multiple Sequence Alignment, Phylogenetic Analysis and Motif Prediction

Based on [11], five plant species *(A. trichopoda*, *E. grandis*, *P. trichocarpa*, *P. abies*, *A. thaliana*) were selected to be used as a reference. These sequences were obtained from the Phytozome database [62] and were used to identify their orthologs in radiata pine transcriptome. Twelve full length sequences from the transcriptome were selected and considered in addition to the reference ones to perform multiple sequence alignment using ClustalX. A rooted phylogenetic tree was generated by the MEGAX [63] construct Maximum Likelihood and JTT matrix-based model with 5000 bootstrap replications.

The MEME web server [64] was used to identify the conserved motifs (H1 and H2 regions, YH motif).

### 4.3. Cryo-Sectioning

Radiata pine stem cuts of 2 cm long were obtained from the control (without inclination) and inclined plants (45°) after 2.5 h, 10 h and 24 h of bending; lower and upper stem sides were clearly named following the strategy reported by Herrera [65]. Stem cuts were embedded in an optimum cutting temperature (OCT) compound (cat #62550-01; Electron Microscopy Sciences, Hatfield, PA, USA) and frozen at −18 °C in a CM1510S Cryostat (Leica, IL, USA). The frozen block with the sample was trimmed, and thick sections were taken and sectioned until the region of interest was reached. Sections (50 µm) containing the intact plant material were cut and placed onto the adhesive side of a cryo-compatible clear adhesive tape (cat #62800-72S; Electron Microscopy Sciences). The slides were stored in the dark at 4 °C until imaged. Frozen OCT-embedded stem sections were stored at −80 °C.

### 4.4. AGP Extraction

Young pine seedlings were inclined and samples were taken at 2.5; 10 and 24 h, as described by Ramos et al. [27]. The analysis was performed in triplicate per condition. Frozen tissues (3 g per condition) were ground to a fine powder in liquid nitrogen. Proteins were extracted in 1 mL of 0.1 M Tris-HCl (pH 7.5), 2% PEG 6000 (*w*/*v*), 2% PVPP (*w*/*v*), 0.2 sodium ascorbate for 1 min at 4 °C according to a modified method of Chabannes et al. [66]. The crude extract was clarified by centrifugation (13,000 g) for 10 min at 4 °C and the supernatant was collected to obtain fraction A, which contained mostly cytosolic and plasma membrane-bound proteins. Fraction B, which contained cell wall-bound proteins, was then extracted from the remaining pellet with 1 NaCl during one night at 4 °C under vigorous shaking. After centrifugation (13,000 g) for 10 min at 4 °C, the supernatant containing cell wall-bound proteins was recovered [45].

To extract AGPs, 3 g of frozen stem samples were ground in 3 mL of extraction buffer containing 50 mM Tris-HCl (pH 8.0), 10 mM EDTA, 2 mM Na_2_SO_5_, and 1% Triton X-100 (*v*/*v*), homogenized by vortexed for 10 min, then incubated at 4 °C for 2.5 h and centrifuged at maximum speed for 10 min in a microcentrifuge. The supernatant was mixed with three volumes of ethanol and the mixture was incubated overnight at 4 °C. The precipitate was collected by centrifugation at maximum speed for 10 min and then resuspended in 50 mM Tris-HCl (pH 8.0), 0.15 M NaCl, 0.02% sodium azide and sonication for 2 min. The ethanol precipitation step was repeated once before the sample was analyzed by rocket electrophoresis [45]. Rocket gel electrophoresis was run with 1% agarose containing 15 μM β-Yariv reagent [66,67,68]. The gel and running buffer consisted of 25 mM Tris and 200 mM glycine (pH 8.4). After completion of electrophoresis, gels were washed overnight with 2% (*w*/*v*) NaCl and dried onto filter paper.

The relative amounts of AGP proteins of stem samples were estimated through rocket electrophoresis, using 1% agarose gels containing 15 μg mL^−1^ of β-glycosil Yariv reagent. Arabic gum was employed as standard at concentrations ranging from 0.5 to 2 μg [45]. The extract is a branched polyglycosylated product.

### 4.5. Immunolabelling and Staining

We used three monoclonal antibodies: JIM7 [69], LM2 [3] and LM6 [33]. Sections were separated in the block with a special pencil to classified control versus samples with inclination. We used samples for triplicate per antibody with four or more stain samples; the same method was used for the three antibodies. Blocking solutions were swapped with 15 μL 1:36 dilutions of supplied antibody solutions; then, sections were incubated at 4 °C for 16 h. Sections were washed twice in 100 μL 1× TBST buffer, either 15 μL of 2 μg μL^−1^ Alexa Fluor™ 488 donkey anti-rat IgG (H + L) (Invitrogen) or 15 μL of 2 μg μL^−1^ Alexa Fluor™ 488 goat anti-mouse IgG (H + L).

### 4.6. Confocal Microscopy

Sample images using confocal microscopy were acquired by a Zeiss LSM 710 attached to an Axio Examiner (Carl Zeiss, Jena, Germany) using a C-Apochromat 40× NA 1.2 water immersion objective lens. The 405 nm and 488 nm diode lasers were used with a 405/488/561 nm main beam splitter.

Three biological replicates were used and, for each of them, at least ten technical replicates; the best three confocal photographs from each sample were processed for antibody staining.

### 4.7. Quantitative PCR Expression Analysis

Total RNA was isolated from *radiata pine* seedlings, base and medium cut from the stem, and these samples were divided facing the upper and lower side. Plants were treated as inclined to 45° in the control, and taken at 2.5, 10 and 24 h. Their stems were divided in different zones, pooled, and immediately frozen in liquid nitrogen and stored at −80 °C until RNA extraction. Total RNA was extracted from 100 mg of frozen tissue using the CTAB extraction procedure described previously [70]. Remaining traces of DNA were removed with DNase I (Biolabs, London, UK) according to the manufacturer’s instructions. Concentration was estimated by an Epoch 2, Take 3 (Agilent technologies, Santa Clara, CA, USA). Primers for quantitative real time-PCR (RT-qPCR) were designed using Primer-BLAST (Table 2), and Housekeeping genes PrUBC2 and PrUBC7 [71]. All primers used in this work are listed in Table 2. SYBR Green/ROX quantitative PCR (qPCR) Master Mix (2×; Fermentas Life Science, Foster City, MA, USA) was used for all qPCR quantifications in a final volume of 20 μL, following the manufacturer’s protocol. All experiments were run on a real-time Mx3000P PCR detection system (Stratagene, San Diego, TX, USA). The cDNA template for each sample was synthesized using 1 μg of DNase-treated total RNA, using a first-strand cDNA synthesis kit (Fermentas Life Science, MA, USA) according to the manufacturer’s instructions. The first-strand RT reaction product was diluted ten-fold, and 2 μL was used for each qPCR reaction. The instrument was set to measure dye florescence at the end of each cycle at the 60 °C annealing/extension step and a melting curve was performed at the end of each reaction. Two-way ANOVA-LSD post hoc was used to determine the main effects of inclination and time of inclination exposure effect for each gene using MaxStatPRO v. 3.6. Significant differences were inferred at * *p*  ≤  0.05, ** *p*  ≤  0.01, *** *p*  ≤  0.001.

## 5. Conclusions

The identification of AGPs and their subsequent classification were based on characteristic domains and motifs. The presence of a GPI anchor would only indicate whether these proteins were anchored to the membrane. The phylogeny showed that four sequences corresponded to group A of fascilin-like (PrFLA1-PrFLA2-PrFLA3-PrFLA4), which were mainly expressed in stems. Histological sections and immunolabeling with JIM7, LM2, and LM6 antibodies showed the presence of AGPs in xylem and highly accumulated in the lower stem side. Xylem cells accumulate AGP proteins in the cell wall in response to inclination.

## Figures and Tables

**Table 1:** Classification group according to phylogeny and other features of glycoproteins.

Gene	Group Length (aa)	AP/PA/SP/TP/GP/VP Repeats in AGP Region	Total PAST%	Part PAST%	Part Length (aa)	Fasciclin Domain	Predicted Signal Peptide	Predicted GPI Anchor	*A. thaliana* FLA BLASTP Hits	Intraspecies FLA BLASTP Hits	Hmmscan
PrFLA1	A/273	6-5-2-1-0-1	0.38	0.08	19 and 8	1	Yes	No	AtFLA11	FLA1(Pinus taeda)	SP-FAS-Dis-TM
PrFLA2	A/264	3-4-1-2-0-0	0.41	0.09	16 and 8	1	Yes	Yes	AtFLA6	p14A9 (Pinus taeda)/arabinogalactan-like protein (Pinus armandii)	SP-FAS-Dis-TM
PrFLA3	A/127	2-2-3-0-0-0	0.48	0.086	11	1	None	Yes	AtFLA6	FLA7(Pinus taeda)	Dis-Dis-TM
PrFLA4	A/164	4-4-1-0-0-1	0.45	0.08	15	1	None	Yes	AtFLA6	FLA7-11(Pinus taeda)-FLA7 (Cinnamomum micranthum f. kanehirae)-AGP-like (Pinus densata)	FAS-Dis
PrFLA5	B/461	4-2-0-0-2-0	0.24	0.04	26	2	Yes	None	AtFLA17	FLA16(Amborella trichopoda)-FLA17(Elaeis guineensis)-FLA17(Elaeis guineensis)-FLA17(Populus trichocarpa)	SP-FAS-Dis-Dis-FAS-Dis
PrFLA6	B/434	4-2-0-0-2-0	0.28	0.25	14	2	None	None	AtFLA17	FLA16(Amborella trichopoda)-FLA16-17(Dendrobium catenatum)-FLA17(Carica papaya)	SP-FAS-Dis-Dis-FAS-Dis
PrFLA7	B/411	9-7-3-2-1-0	0.38	0.09	8 and 42	2	Yes	Yes	AtFLA17	FLA8(Pinus taeda)-FLA10(Hevea brasiliensis)-FLA10(Citrus clementina)	SP-FAS-FAS
PrFLA8	B/221	0-0-0-0-0-0	0.27	0	0	1	None	None	AtFLA17	FLA7-11(Pinus taeda)-FLA7 (Cinnamomum micranthum f. kanehirae)-AGP-like (Pinus densata)	SP-FAS-Dis
PrFLA9	C/845	13-9-3-2-3-0	0.33	0.06	8, 41 and 14	3	None	None	AtFLA8	FLA8(Pinus taeda)-FLA16(Amborella trichopoda)-FLA16(Dendrobium catenatum)	SP-FAS-Dis-TM
PrFLA10	C/390	5-6-1-2-0-1	0.36	0.09	44	2	Yes	None	AtFLA8	FLA8(Pinus taeda)-FLA10(Populus alba)-FLA10(Populus trichocarpa)-FLA8(Prunus avium)	SP-FAS-Dis-TM
PrFLA11	D2/464	4-2-1-0-0-1	0.34	0.03	4, 5 and 8	2	Yes	None	AtFLA4	FLA4 isoform X1(Amborella trichopoda)-FLA4(Nymphaea colorata)-FLA4(Glycine soja)	SP-FAS-FAS-Dis-Dis-TM
PrFLA12	D2/415	3-4-5-1-0-0	0.36	0.05	7 and 14	2	None	None	AtFLA4	FLA4 isoform X1(Amborella trichopoda)-FLA4(Nymphaea colorata)-FLA4(Elaeis guineensis)	FAS-FAS-Dis

**Table 2:** Primer list.

Gene	Fordward	Reverse
PrFLA1	GATAGGATTTATTGGGGCAATTCAC	CTGCCCCTATAAATCAGAATTCCAT
PrFLA2	TAGCGCCCGTCGTTTAAATG	CTGACGCAACGATTACTTACCAAAT
PrFLA3	AGCACCAGCACCAGCACCAGTCTT	GGGAAATGCAATGGGCCAA
PrFLA4	AGCCAATCTGACACAACTGCTATCA	CAGGATCAGATGGTGCAAATATTGT

## Data Availability

Not applicable.

## Supplementary Materials

The following supporting information can be downloaded at: https://www.mdpi.com/article/10.3390/plants11091190/s1, Supplementary Figure S1. Alignment of radiata pine versus Arabidopsis thaliana sequences, group A according to phylogeny. Highlighting whether there is the presence of FAS domains (mustard-colored line) and whether the elements of this domain contain their characteristic regions and motifs, H1 (blue square), YH (green square) and H2 (yellow square); Supplementary Figure S2. Alignment of radiata pine versus Arabidopsis thaliana sequences, group B according to phylogeny. Highlighting whether there is the presence of 1 or 2 FAS domains (mustard-colored line) and whether the elements of this domain contain their characteristic regions and motifs, H1 (blue square), YH (green square) and H2 (yellow square); Supplementary Figure S3. Alignment of radiata pine versus Arabidopsis thaliana sequences, group C according to phylogeny. Highlighting whether there is the presence of 1 or 2 FAS domains (mustard-colored line) and whether the elements of this domain contain their characteristic regions and motifs, H1 (blue square), YH (green square) and H2 (yellow square); Supplementary Figure S4. Alignment of radiata pine versus Arabidopsis thaliana sequences, group D according to phylogeny. Highlighting whether there is the presence of 1 or 2 FAS domains (mustard-colored line) and whether the elements of this domain contain their characteristic regions and motifs, H1 (blue square), YH (green square) and H2 (yellow square); Supplementary Figure S5. Measurement of area of staining in samples treated with Antibody. (A) Staining area treated with JIM7 antibody, in control stem samples, inclined at 2.5 h upper and lower cuts, 10 h upper and lower cuts, 24 h upper and lower cuts. (B) Staining area treated with LM2 antibody, in control stem samples, inclined at 2.5 h upper and lower cuts, 10 h upper and lower cuts, 24 h upper and lower cuts. (C) Staining area treated with LM6 antibody, in control stem samples, inclined at 2.5 h upper and lower cuts, 10 h upper and lower cuts, 24 h upper and lower cuts. The letters indicated significance differences, and * *p* < 0.05, ** *p* < 0.01, *** *p* < 0,001; Table S1. Putative FLAs identified in Plants.
