# Supplementary figures and images for: Differential Expression of Arabinogalactan in Response to Inclination in Stem of Pinus radiata Seedlings
Source: Plants (Basel). 2022 Apr 28;11(9):1190. doi: 10.3390/plants11091190 (PMC9104628; doi:10.3390/plants11091190)

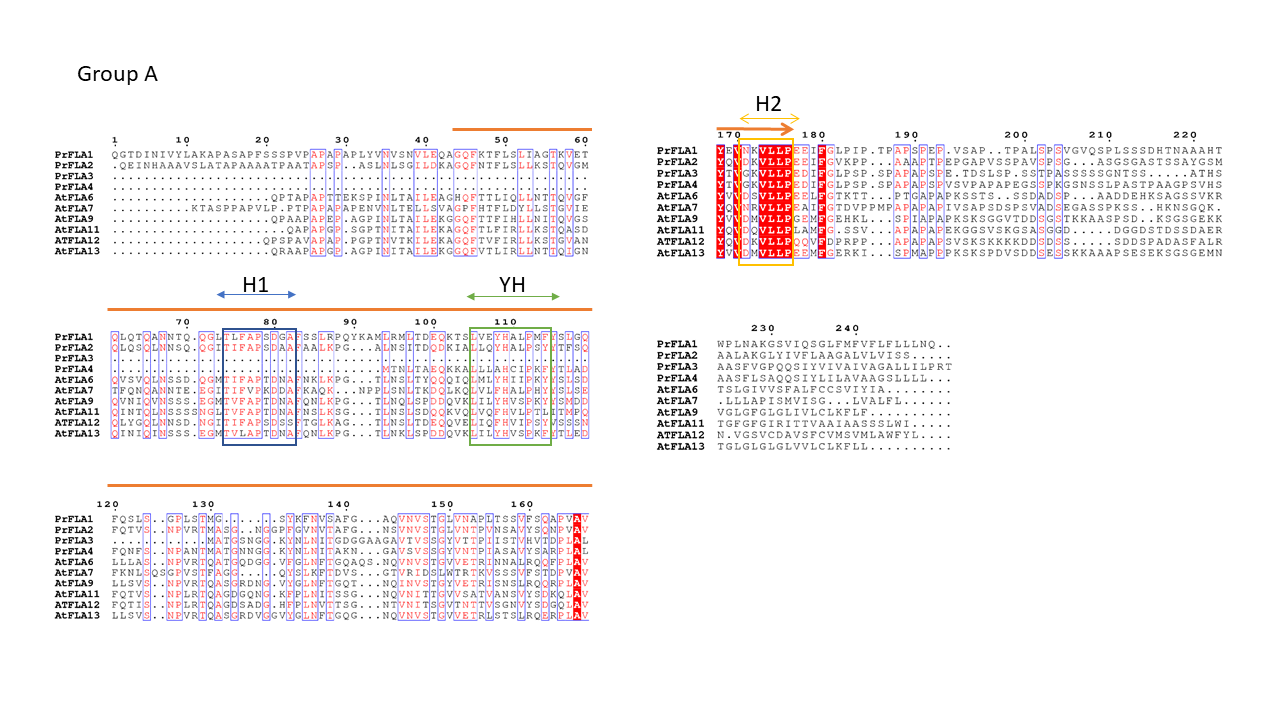

Supplement: Supplementary file 1 [file plants-11-01190-s001.zip › supplementary figure 1.TIF]

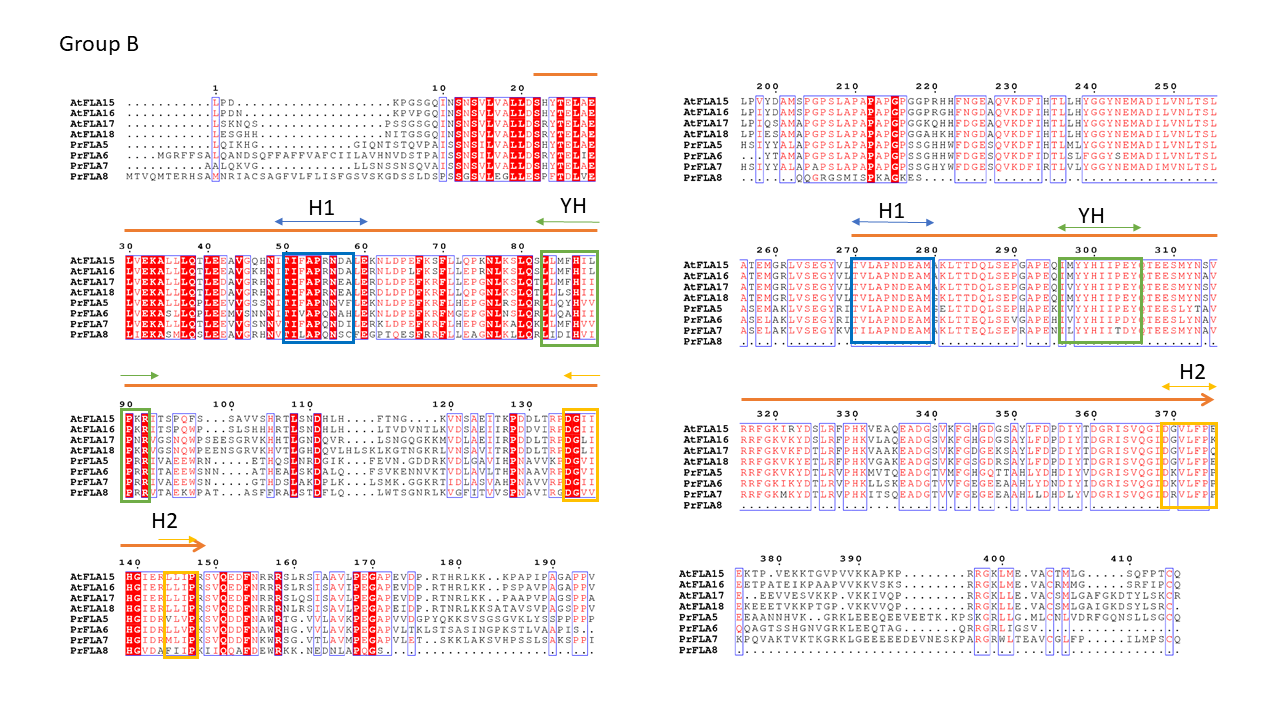

Supplement: Supplementary file 1 [file plants-11-01190-s001.zip › Supplementary figure 2.TIF]

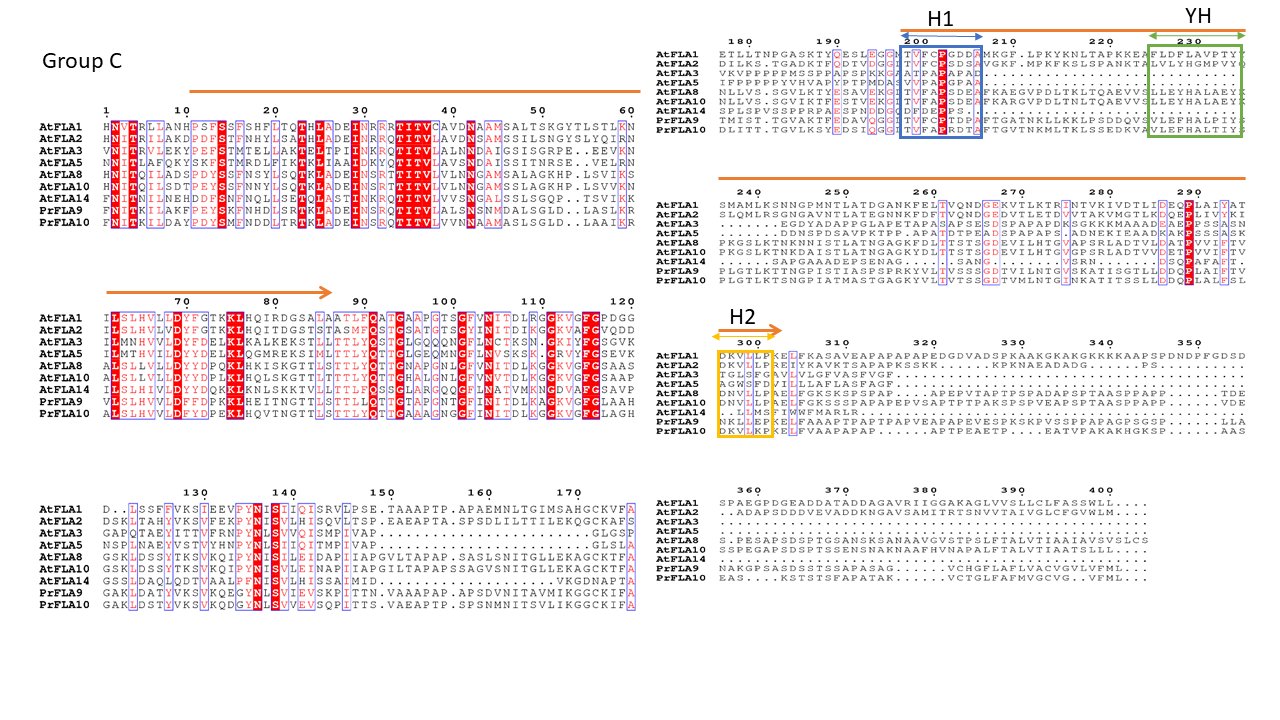

Supplement: Supplementary file 1 [file plants-11-01190-s001.zip › Supplementary figure 3.TIF]

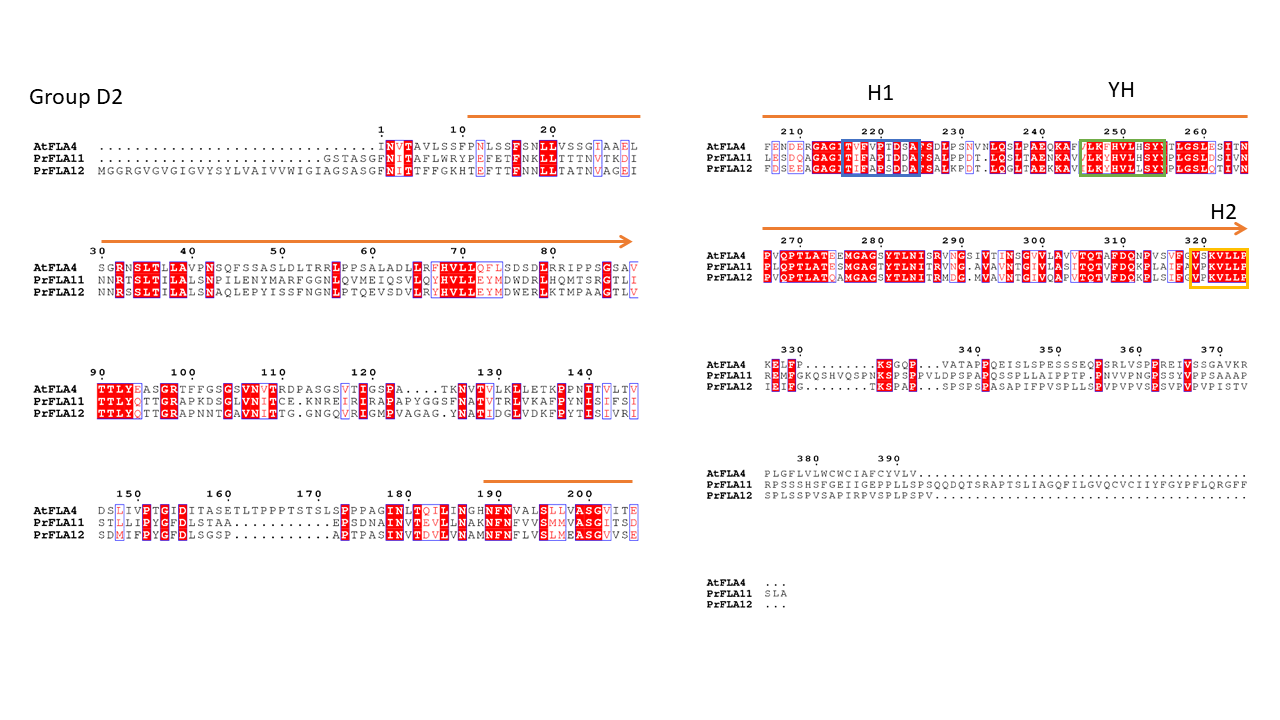

Supplement: Supplementary file 1 [file plants-11-01190-s001.zip › Supplementary figure 4.TIF]

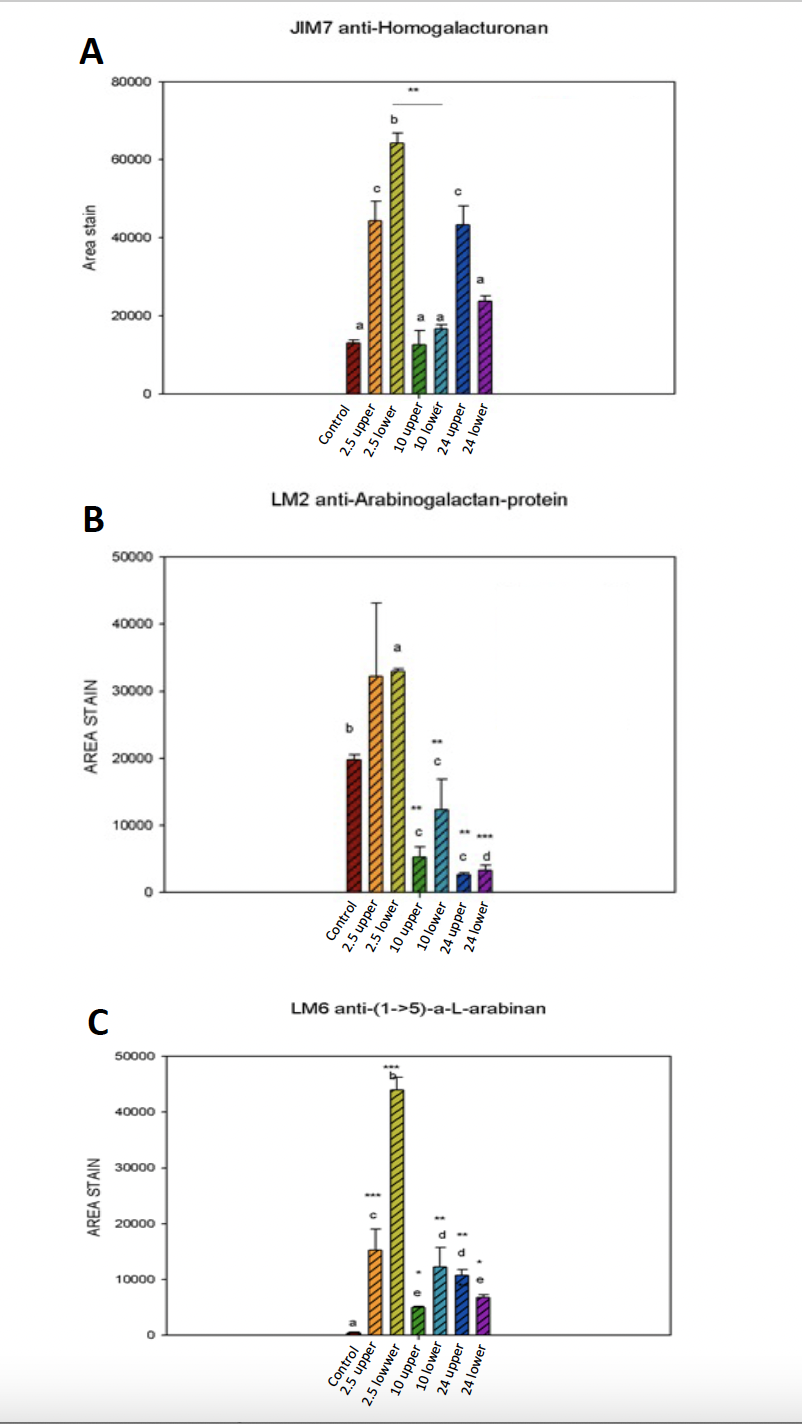

Supplement: Supplementary file 1 [file plants-11-01190-s001.zip › Supplementay figure 5.png]
